# Supplementary material for: Confinement of a Styryl Dye into Nanoporous Aluminophosphates: Channels vs. Cavities
Source: Int J Mol Sci. 2024 Mar 22;25(7):3577. doi: 10.3390/ijms25073577 (PMC11011965; doi:10.3390/ijms25073577)
Supplement: Supplementary file 1 [file ijms-25-03577-s001.zip › ijms-2890201-supplementary.pdf]

Electronic supporting information for:

Confinement of a styryl dye into nanoporous aluminophos-phates: channels vs cavities

by Ainhoa Oliden-Sánchez,[1] Rebeca Sola-Llano,[1] Joaquín Pérez-Pariente,[2] Luis Gómez-Hortigüela, [2,*] and Virginia Martínez-Martínez,[1,*]

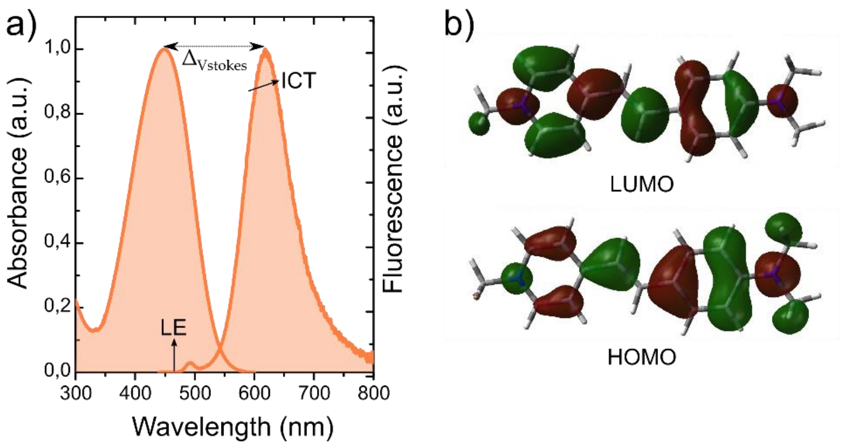

Figure S1. a) Height-normalized absorption and emission spectra (λ_{exc} = 420 nm) recorded for the 4-DASPI dye in aqueous solution; b) HOMO-LUMO orbitals of 4-DASPI dye.

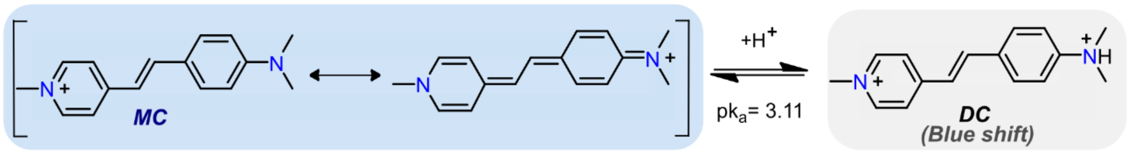

Figure S2. Equilibrium between monocation (MC) and dication (DC) species of 4-DASPI dye in aqueous solution.

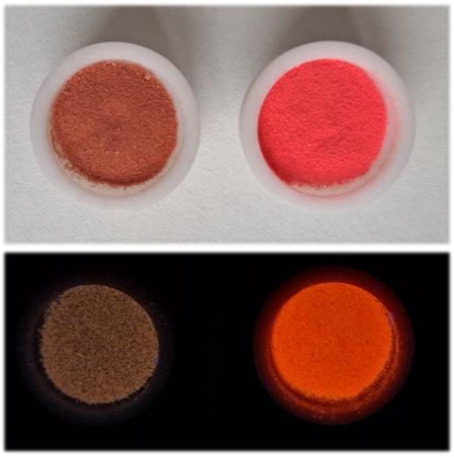

Figure S3. Photographs of samples 4-DASPI@MgAPO-AEL (1) and (6) in powder under ambient and UV light.

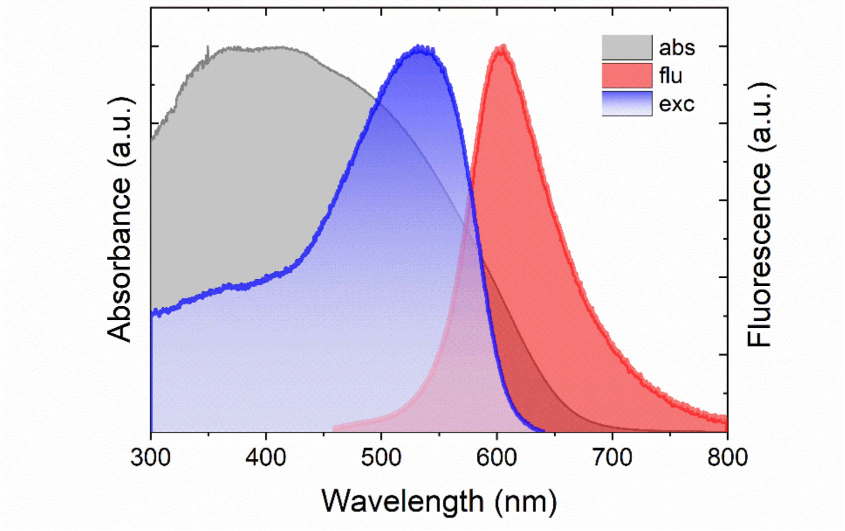

Figure S4. Absorption, excitation and emission (λ_{exc} = 450 nm) spectra of sample 4-DASPI@MgAPO-AEL-1

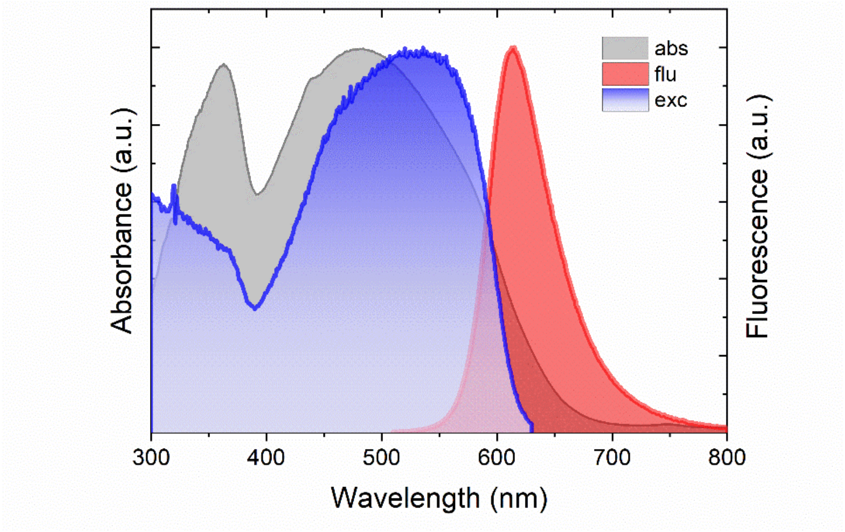

Figure S5. Absorption, excitation and emission (λ_{exc} = 500 nm) spectra of sample 4-DASPI@MgAPO-AEL-6 .
